# Evaluation of Product Distribution in Chemostat and Batch Fermentation in Lactic Acid-Producing *Komagataella phaffii* Strains Utilizing Glycerol as Substrate

**DOI:** 10.3390/microorganisms8050781

**Published:** 2020-05-22

**Authors:** Nadielle Tamires Moreira Melo, Gabriela Coimbra Pontes, Dielle Pierotti Procópio, Gabriel Caetano de Gois e Cunha, Kevy Pontes Eliodório, Hugo Costa Paes, Thiago Olitta Basso, Nádia Skorupa Parachin

**Affiliations:** 1 Pós-Graduação em Ciências Genômicas e Biotecnologia, Universidade Católica de Brasília, Brasília, DF CEP 70790-160, Brazil; nadytamires@gmail.com; 2 Grupo de Engenharia de Biocatalisadores, Departamento de Biologia Celular, Instituto de Ciências Biológicas Bloco K, Universidade de Brasília (UnB), Campus Darcy Ribeiro, Brasilia, Federal District 70.790-900, Brazil; gabicoimbrap@gmail.com; 3 Department of Chemical Engineering, Escola Politécnica, University of São Paulo, São Paulo-SP 05508-010, Brazil; diellepierotti@gmail.com (D.P.P.); gabrielcgc@usp.br (G.C.d.G.eC.); kevypontes@usp.br (K.P.E.); thiagobasso@usp.br (T.O.B.); 4 Clinical Medicine Division, University of Brasília Medical School, Darcy Ribeiro Campus, Asa Norte, Brasília, Federal District 70910-900, Brazil; sorumbatico@gmail.com

**Keywords:** *K. phaffii*, lactic acid, arabitol, arabitol dehydrogenase, crude glycerol, chemostat cultivation

## Abstract

Lactic acid is the monomeric unit of polylactide (PLA), a bioplastic widely used in the packaging, automotive, food, and pharmaceutical industries. Previously, the yeast *Komagataella phaffii* was genetically modified for the production of lactate from glycerol. For this, the bovine L-lactate dehydrogenase- (LDH)-encoding gene was inserted and the gene encoding the pyruvate decarboxylase (PDC) was disrupted, resulting in the GLp strain. This showed a yield of 67% L-lactic acid and 20% arabitol as a by-product in batches with oxygen limitation. Following up on these results, the present work endeavored to perform a detailed study of the metabolism of this yeast, as well as perturbing arabitol synthesis in an attempt to increase lactic acid titers. The GLp strain was cultivated in a glycerol-limited chemostat at different dilution rates, confirming that the production of both lactic acid and arabitol is dependent on the specific growth rate (and consequently on the concentration of the limiting carbon source) as well as on the oxygen level. Moreover, disruption of the gene encoding arabitol dehydrogenase (ArDH) was carried out, resulting in an increase of 20% in lactic acid and a 50% reduction in arabitol. This study clarifies the underlying metabolic reasons for arabitol formation in *K. phaffii* and points to ways for improving production of lactic acid using *K. phaffii* as a biocatalyst.

## 1. Introduction

Lactic acid is a bio-product with a consolidated market. It is the monomeric unit for the production of polylactide (PLA), a biopolymer that has garnered great interest as a biodegradable biological substitute for conventional plastics of petrochemical origin [1,2]. It has been estimated that the cost of producing lactic acid must be reduced by 50% for PLA to be competitive in the market [3]. The most commonly used bacteria for lactic acid production are lactobacilli that make it naturally, such as *Lactobacillus helveticus*, *Lactobacillus lactis*, and *Lactobacillus plantarum* [4,5]. However, they have both L-lactate dehydrogenase (L-LDH) and D-lactate-dehydrogenase (D-LDH), generating a racemic mixture that is poorly suited for PLA synthesis. In view of the growing interest in the development of sustainable technologies, the use of genetic engineering in the manipulation of microorganisms for lactic acid production has been an area of intense activity [6,7,8]. Among considered organisms, yeasts stand out against bacteria as they are more adapted to industrial conditions such as pH and pressure fluctuations [9]. The yeast *Komagatataela phaffii* (formerly *Pichia pastoris*) has been of interest for the production of lactic acid, mainly due to its ability to consume glycerol, a plentiful carbon source with low added value. This is considered advantageous as a way to make the biodiesel production chain, of which glycerol is a by-product, more profitable, and to maximize the use of biomass contributing to the biorefinery concept [10,11,12,13]. Several studies demonstrate the efficient use of crude and/or pure glycerol as a carbon source by *K. phaffii* in the production of heterologous proteins, industrial enzymes and high added-value chemicals [10,14,15,16].

In a previous report, we introduced the gene of the bovine L-LDH (bLDH) into a strain of *K. phaffii*, along with engineered overexpression of two transporters—PAS (a *K. phaffii* putative lactate transporter) and JEN1p (an *S. cerevisiae* lactate transporter) [14]. Subsequently, we deleted the gene encoding the pyruvate decarboxylase (PDC) of this yeast with a view to blocking the loss of carbon from lactic fermentation to ethanol and acetate production, based on previous work on the deletion of PDC in *S. cerevisiae* [17,18,19,20]. The latter strain, named GLp, achieved a lactic acid yield of approximately 0.67 and a productivity of 0.15 g∙L^−1^∙h^−1^, thus showing that this yeast has a potential for producing lactic acid using crude glycerol [21]. However, the fermentation data indicated that the process resulted in the production of arabitol (C_5_H_12_O_5_; Ara), in an amount that suggests that the failure to achieve higher yields of lactic acid are explained by the appearance of this undesirable by-product [21]. At low oxygen availability, a previous study showed that the reduced oxygen availability for the electron transport chain leads to higher NADH/ NAD cytosolic ratios [22]. In the GLp strain, this is intensified in the conversion of glycerol to lactic acid where one mol of NADH is formed per mol of glycerol consumed. We hypothesized that in the GLp strain, this imbalance is compounded by the absence of the PDC gene, thus removing the possibility of re-oxidizing NADH to NAD+ by the formation of ethanol or acetate. The depletion of NAD+ would cause the metabolic flow from glycerol to pyruvate to slow down, leading GA3P/DHAP to flow in reverse and into the pentose phosphate pathway (PPP), at the end of which xylose or ribulose reduction into arabitol is coupled with the oxidation of NADH, thus relieving the redox imbalance caused by glycerol fermentation to lactic acid [21]. 

In other words, although the process is viable as a source of energy for the yeast, the low yield is due to a metabolic imbalance that cannot be solved with adjustments in the fermentation conditions. In a highly productive strain of *Escherichia coli* engineered and evolved to convert crude glycerol to D-lactate, a similar redox imbalance is ultimately corrected by coupling the process to nitrate reduction [6], lending credence to our hypothesis that arabitol production in the GLp strain occurs in response to the same problem. Therefore, this work aimed at cultivating GLp strain in chemostats and in fed-batch mode with different dilution and oxygenation rates. Finally, a new genetic modification was introduced in Glp strain by disrupting the gene that encodes its putative arabitol dehydrogenase (ArDH), the enzyme that catalyzes the final step of the PPP that regenerates NAD+. This system-level study helped to understand the physiological adaptations of cellular mechanisms to low oxygen availability in a recombinant strain of *K. phaffii* for the production of lactic acid. 

## 2. Materials and Methods 

### 2.1. Strains and Plasmids 

All plasmids and strains used in this study are listed in Table 1, and were kept in cryovials with 20% glycerol at −80 °C. The *E. coli* strains used during the cloning steps were grown at 37 °C in lysogeny broth (0.5% yeast extract, 1% peptone, and 0.5% sodium chloride) supplemented with ampicillin (100 μg/mL). The *K. phaffii* strains were grown at 30 °C in YPD (1% yeast extract, 2% peptone and 2% dextrose) supplemented with geneticin (G418: 500 μg/mL), zeocin (100 μg/mL) and or hygromycin (200 μg/mL) as needed.

### 2.2. Construction of the ArDH Knockout Cassette

**Figure 1:**
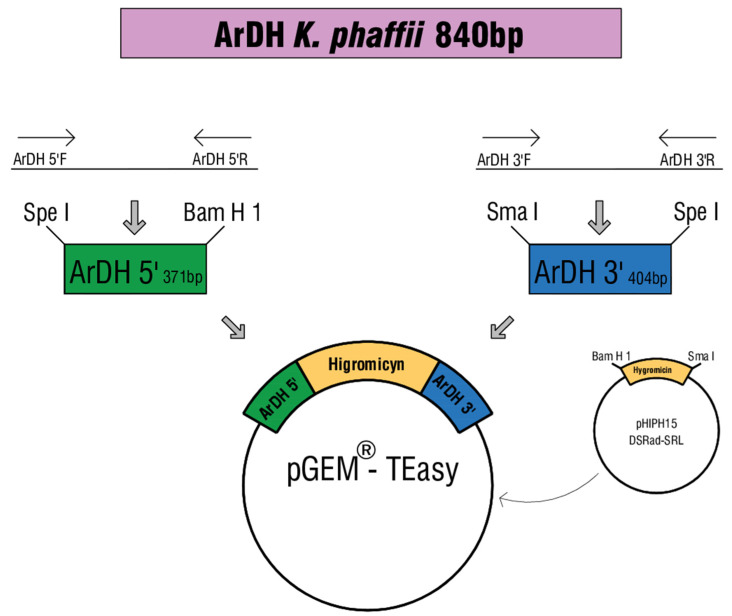
Construction of arabitol dehydrogenase (ArDH) knockout cassette. The entire ArDH-encoding gene has 840 bp and it was divided into two fragments, ArDH’5 e ArDH3′, with 371 and 404 base pairs, respectively. The hygromycin phosphotransferase (hph) cassette was amplified from the vector pHIPH15 DsRed-SKL. Each fragment was cloned into the pGEM plasmid using the indicated restriction enzymes. The final cassette has a total length of 2556 bp.

The strategy used for the construction of the ArDH interruption cassette is summarized in Figure 1. Once the DNA sequence for the ArDH (EC1.1.1 250) enzyme was obtained, a PCR was performed to amplify the complete ORF. For that, the genomic DNA of *K. phaffii* GS115 was used as a template. The primers used were ArDH 5’F and ArDH 3’R (Table 2) and the amplicon was approximately 840 bp long, corresponding to the expected size. For the interruption of the *ARDH* gene, the sequence was split into two fragments: ArDH 5’ and ArDH 3’ (with 371 and 404 base pairs, respectively - see Figure 1). First, the hygromycin phosphotransferase (*hph*) cassette was amplified from the vector pHIPH15 DsRed-SKL [23], with the HIGRO 5’F and HIGRO 3’R primers (Table 2) resulting in an amplicon of 1781 bp, which was cloned into the pGEM^®^ T Easy vector. The insertion of the ARDH5’ fragment was done using SphI and BamHI after amplification by PCR using the ARDH5’ F and ARDH5’ R primers (Table 2). After confirmation by PCR, the new construct was treated with SmaI and SpeI to insert the ARDH3′ amplicon digested with the same enzymes downstream of the hygromycin resistance marker. PCR amplifications were performed in a reaction volume of 20 μL containing 2.5 pmol of each primer, 2.5 units of Taq DNA polymerase (Sinapse Biotecnologia, Tatuapé, Brazil), 0.2 μM of each dNTP, 2.0 μL of 10× reaction buffer (10 mM Tris-HCl pH 8.3, 50 mM KCl, 1.5 mM MgCl_2_) and 50 ng of DNA template. The resulting plasmid was named pGEMHY ardh (Table 1) and contained a cassette composed of ARDH5’, followed by the *hph* cassette and ARDH3’, for a total length of 2556 bp.

### 2.3. ArDH Knockout in K. phaffii 

The ArDH deletion cassette was introduced into the *K. phaffii* GLp strain (Table 1) following the protocol described previously [21]. Briefly, a single colony was inoculated into 25 mL of YPD medium, and after 24 h at 28 °C and 250 rpm, 1 mL of cells was used to inoculate 100 mL of YPD medium. When the OD_600_ reached approximately 1.5, the cells were harvested by centrifugation and resuspended in 50 mL of cold and sterile water. This wash step was repeated twice, followed by a resuspension in 5 mL of cold 1 M sorbitol. All centrifugation steps were performed at 1500× *g* for 5 min at 4 °C. Finally, the cells were re-suspended in 150 μL of cold 1 M sorbitol and 80 μL were mixed with 5–10 μg DNA. This suspension was then loaded into a 2 mm electroporation cuvette (Bio-Rad, Berkeley, CA, USA) and incubated on ice for 5 min. Electroporation settings were 7.5 kV∙cm^−1^, 400 Ω, and 25 μF. Immediately after the pulse, 1 mL of cold 1 M sorbitol was added and the cells were incubated at 30 °C for three hours. At last, the cells were plated onto YPD agar supplemented with hygromycin (200 μg∙mL^−1^) and incubated at 30 °C for three days. This resulted in the GLpard strain (Table 1). To confirm the *ARDH* deletion in *K. phaffii*, 30 colonies were investigated using PCR amplification using the ArDH5’ F and ArDH 3’. The amplification of a fragment size of 2556 bp indicated the insertion of the cassette. 

### 2.4. Cultivation in Bioreactor

#### 2.4.1. Medium 

For all cultivations in bioreactors, the UAB defined medium was utilized as previously describe [21], with modifications. The composition (per liter) of the medium was: 1.8 g/L Citric Acid (C_6_H_8_O_7_); 0.02 g/L Ca Hydrochloride Dihydrate (CaCl_2_·2H_2_O); 12.4 g/L dibasic ammonium phosphate ((NH_4_)_2_HPO_4_); 0.5 g/L Magnesium sulfate heptahydrate (MgSO_4_·7H_2_O); 0.9 g/L Potassium chloride (KCl); pH 5.0, adjust with HCl; with 25% HCl. PTM1 trace salts stock solution (per liter) was composed of 6.0 g/L Copper sulphate pentahydrate (CuSO_4_·5H_2_O); 0.08 g/L Sodium iodide (NaI); 3.0 g/L Manganese sulfate monohydrate (MnSO_4_·H_2_O); 0.2 g/L Sodium molybdate dihydrate (Na_2_MoO_4_·2H_2_O); 0.02 g/L Boric acid (H_3_BO_3_); 0.5 g/L Cobalt chloride (CoCl_2_); 20.0 g/L Zinc chloride (ZnCl_2_); 65.0 g/L Iron sulfate heptahydrate (FeSO_4_·7H_2_O); 5.0 mL/L Sulfuric acid (H_2_SO_4_—95%−98%), 0.2 mg.L^−1^ biotin, pH was adjusted to 5.0 with 25% HCl and 0.2 mL/L of antifoam glanapon 2000 (Konc, Bussetti, Vienna, Austria) was added.

#### 2.4.2. Chemostat Cultivations

Chemostat cultivations of the GLp strain were carried out in a 2.0 L water-jacketed Labfors 5 bioreactor (Infors AG, Basel, Switzerland) with a 1.0 L working volume kept constant by a mechanical drain controlled by a peristaltic pump. Chemostat cultures were performed for at least five residence times (τ) prior to sampling. The pH was adjusted to 5.0 with 25% HCl and 0.2 mL·L^−1^ of antifoam glanapon 2000 (Konc, Bussetti, Vienna, Austria) was added. A starter culture from a single GLp colony was generated by incubation overnight at 30 °C, 200 rpm in a 0.5-L conical baffled flask containing 50 mL of minimal medium with glycerol as the carbon source and supplemented with biotin (10 g·L^−1^ yeast nitrogen base, 0.2 mg·L^−1^ biotin, 10 g·L^−1^ glycerol). The bioreactor was inoculated from the starter culture at an initial OD_600_ of 0.3–0.5. After complete glycerol consumption (monitored by a sharp drop in the CO_2_ concentration in the off-gas stream), continuous cultures were started at different dilution rates (0.05 h^−1^, 0.10 h^−1^, and 0.15 h^−1^) with 21% oxygen concentration in the inlet gas stream (normoxia). Then, dilution rate was set to 0.05 h ^−1^ with an oxygen limitation (4% oxygen concentration in the inlet gas stream), resulting in a hypoxic condition. The aeration rate was set to 1 vvm and the offgas O_2_ and CO_2_ concentrations were measured by an off-gas analyzer. Temperature was maintained at 30 °C, the stirring rate was 600 rpm and a pH value of 5.0 was controlled by automated injection of a 15% (*v/v*) ammonia solution. All chemostat cultures were carried out in a way to minimize oxygen diffusion into the system, using Viton “O”-rings and Norprene^®^ tubing. The dissolved oxygen concentration was monitored constantly by an oxygen electrode (Mettler-Toledo, Columbus, OH, USA).

All experiments were conducted in duplicate, and the presented values shown are the average of the replicates ± the deviation of the mean. Steady state was assumed when five-volume changes had passed since the last perturbation in the cultivation conditions, and the culture dry weight and the specific carbon dioxide production rate varied by less than 2% over two volume changes. 

#### 2.4.3. Batch Fermentation 

A 100 mL starter culture was prepared with 20 g·L^−1^ glycerol in minimal medium with biotin as above and was grown for approximately 30 h at 30 °C and 200 rpm. We used a BioFlo 115 3 L BioFlo 115 bioreactor (Eppendorf AG, Hamburg, Germany) to set one-liter cultures at an initial OD_600_ of 2, and starting with glycerol concentrations of 20, 60 or 80 g·L^−1^. All batch experiments were performed at 30 °C, 600 rpm, dissolved oxygen at 50%, and pH set to 5 and controlled with 3 M NH_4_OH. Samples were collected every 3 h and centrifuged at 12,000 g for 5 min. The supernatant was stored at −20 °C for high-performance liquid chromatography (HPLC) analysis.

#### 2.4.4. Fed-Batch Fermentations

Fed-batch fermentation was performed as previously reported [21] but changing the glycerol concentration during the oxygen limitation phase. Three concentrations of glycerol were tested: 20, 60 or 80 g·L^−1^ in a single pulse for each fermentation. The samples were collected every 3 h and centrifuged at 12,000 g for 5 min. The supernatant was stored at −20 °C for HPLC analysis.

### 2.5. Biomass Determination

Cell density was monitored by optical density at 600 nm. Dry cell weight (DCW) was measured gravimetrically in duplicate. Briefly, a known volume of sample (5–10 mL) was filtered through dry, pre-weighed 0.45 μm polyether sulfone filters (Frisenette, Knebel, Denmark) and washed with distilled water. Filters were dried in a microwave oven at 150 W for 20 min, cooled in a desiccator for at least two hours, and then weighted. The DCW was the difference between the recorded weights before and after filtration. 

### 2.6. Substrate Consumption and Cellular Products Quantification 

Glycerol, lactic acid, acetic acid, ethanol, and arabitol were quantified using HPLC (Shimadzu, Kyoto, Japan) equipped with UV (210-nm) and refractive index detectors as previously described [21]. A pre-column Guard Column SCR (H) (50 mm × 4 mm id) with stationary-phase, sulfonated styrene-divinylbenzene copolymer resin was used. The chromatography flow rate was 0.6 mL·min^−1^ and the injection volume was 20 μL, using a Shim-pack SCR-101H (Shimadzu; 300 mm × 7.9 mm id) column equilibrated at 60 °C with 5 mM H_2_SO_4_ as the mobile phase. 

### 2.7. Statistical Analysis

Chemostat cultivation data were checked for consistency using elemental mass balances and common reconciliation procedures [24]. The biomass molecular formula used was selected according to the specific biomass composition of *K. phaffii* growing on glycerol. In all the cultures, a statistical consistency test was applied with a confidence level of 95%. There was no evidence of gross measurement errors.

## 3. Results and discussion

### 3.1. Effect of the Dilution Rate and Oxygen Concentration for the Production of Lactic Acid in Glycerol-Limited Chemostat Cultures

Recently, there has been great interest in the development of continuous lactic acid fermentation due to its potential to increase lactic acid titers and reduce production costs, in addition to its potential to foster our understanding of non-conventional yeast physiology [7]. In order to understand in which conditions arabitol was formed as by-product, the GLp strain was cultured in glycerol-limited chemostat at different dilution rates and oxygenation conditions. All chemostat data are summarized in Table 3. In full aerobic conditions (21% of oxygen in the inlet gas, resulting in a dissolved oxygen concentration above 50%), three dilution rates were tested, while under oxygen limitation (4% of oxygen in the inlet gas, resulting in a dissolved oxygen concentration below the detection limit), only one dilution rate could be tested since, at higher dilution rates, wash-out was observed.

Under full aerobic conditions (21% of oxygen in the inlet gas), biomass yield during steady state ranged between 0.69–0.74. These values are in the upper range of biomass yields previously reported for glycerol as the limiting C-source [13,25]. In addition to the impact of the specific growth rate on the biomass yield described above, it is also known that other factors, such as the carbon source, have a significant influence on this parameter [26]. However, similar profiles are observed when comparing the biomass yield on glycerol with the biomass yield for cultures limited in glucose [22]. Whereas biomass yield decreased by about 7% with increased dilution rates (Table 3), lactate yield increased 70%, meaning that the higher the availability of the limiting substrate, the higher the shunting of the carbon source to lactate production by the growing yeast. Nevertheless, the yield of lactate in *K. phaffii* in continuous culture is roughly 36% lower than the previous values observed in fed-batch cultivations [21,27]. In addition, the lactate yield was nearly 5 times lower than the ones previously reported in the literature made in other organisms for the production of lactic acid using cheap raw materials such as glycerol in continuous cultivation mode [7].

Irrespective of the oxygen availability during steady-state, ethanol, acetate, pyruvate, and arabitol could not be detected in any of the tested dilution rates. Arabitol is a C5 sugar alcohol related to the pentose phosphate pathway (PPP), which has already been shown to be produced by some yeast species during fermentation [22,28]. For example, the accumulation of glycerol and arabitol was previously observed in *Pichia anomala* cultures during growth in environments with a high salt content and in highly concentrated sugar media [29], suggesting that arabitol has the same physiological role as glycerol in the protection against osmotic stress. Previously, in a glucose-limited chemostat cultivation under normoxia (21% oxygen concentration in the inlet gas stream) using *K. phaffii* strain X-33, arabitol was also not detected. However, when the oxygen concentration was reduced to 11% or 8%, 0.9, and 2.88 g·L^−1^ were detected, respectively, suggesting that the formation of arabitol was oxygen-dependent [27,30]. The disagreement between the different data may be explained by the strain background and by the carbon source utilized (glucose vs. glycerol). Glucose fermentation using wild-type *K. phaffii*, such as X-33 strain, does not generate a redox imbalance since the PPP branching point of glycolysis lies downstream of glucose, which means that part of the carbon might be shunted towards the PPP both as a way to relieve an imbalance from other causes such as amino acid biosynthesis [26,31] or as a source of nucleotide precursors needed for cellular division. In contrast, when glycerol is used as carbon source, it enters glycolysis as GA3P/DHAP and downstream of the branching point, which means it is not readily available for the PPP, unless high lactate production causes a more serious redox imbalance as we hypothesized.

In chemostast cultivations, the highest lactate yield obtained was under oxygen limitation, corresponding to a fivefold increase relative to 21% oxygen (Table 3). *K. phaffii* has a fully respiratory metabolism under normoxic conditions and a shift to fermentative metabolism can be observed under oxygen-limiting conditions and a clearly respiratory metabolism is shown under hypoxic conditions [22,27]. Hence, it can be observed that the formation of lactate depends on the residual concentration of glycerol during steady state in hypoxia, since the residual concentration of glycerol in this state reaches 4.23 g·L^−1^.

### 3.2. Batch Fermentation

**Figure 2:**
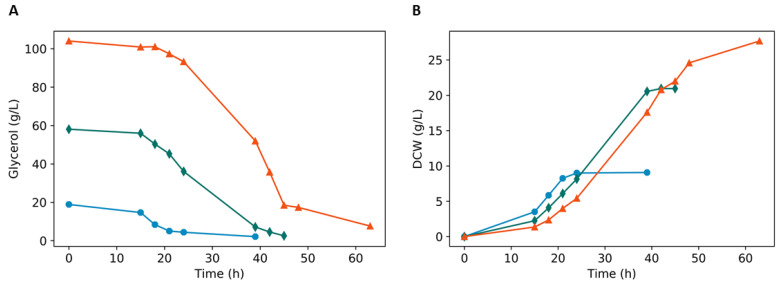
GLp batch aerobic culture showing the consumption of glycerol (**A**), biomass (**B**) in different initial glycerol concentrations: 2% (blue circle), 6% (green diamond), and 10% (orange triangle). All fermentations were with the GLp strain. The experiments were carried out in biological duplicates, and the figure shows a typical fermentation profile.

**Figure 3:**
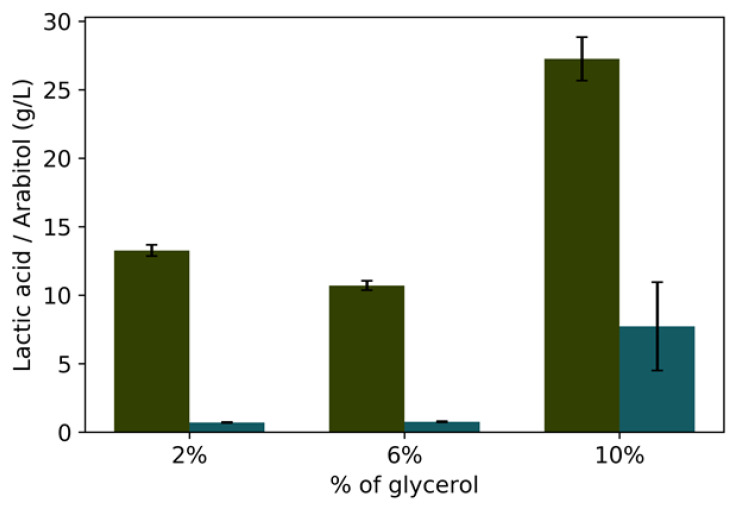
GLp batch aerobic culture on the accumulation of lactate and arabitol in different initial glycerol concentrations: 2%, 6%, and 10%, lactate (green) and arabitol (blue) production. All fermentations were with the GLp strain. The experiments were carried out in biological duplicates.

Since no arabitol was observed in chemostat cultures and it was suggested to be dependent on both the concentration of glycerol and on the rate of lactic acid formation, aerobic batch fermentations were performed with initial glycerol concentrations of 2%, 6%, or 10% (Figure 2A,B, Figure 3 and Table 4) while maintaining oxygenation constant. Higher glycerol concentrations resulted in an increase of biomass and at the end of the fermentations, the highest production of lactic acid and arabitol were with 10% glycerol: the GLp strain produced 24 g·L^−1^ of lactic acid and 7.02 g·L^−1^ of arabitol. These results show that the production of both lactic acid and arabitol is higher the greater the availability of carbon (Figure 3), even in aerobic conditions. 

This suggests that lactate yields can be increased by a bioprocess strategy that overwhelms the yeast respiratory and biomass-producing pathways, causing carbon to “spill over” to lactate production instead of fueling anabolic pathways or energy production. Indeed, this is likely to be the reason why previously not only lactate, but arabitol production by GLp were observed in aerobic conditions and high concentrations of glycerol [21]. Put together, the current and previous data suggest arabitol production, which also rises in tandem with the initial concentration of glycerol, occurs when glycerol is fermented instead of respired, which supports the hypothesis that this is due to a redox imbalance [32], and explains why arabitol appears even in normoxia if the initial concentration of glycerol is high. 

Table 4 shows the results of batch fermentations under normoxia and hypoxia, and different initial concentrations of glycerol. Lactic acid yield is variable in terms of the increase in glycerol as a carbon source. In total, these results show that, under aerobic conditions, the main yeast products are biomass, lactic acid, and arabitol for all concentrations of glycerol. Although the yield of lactic acid does not follow a clear pattern in aerobic culture relative to the initial concentration of glycerol (see also Figure 3), the production of arabitol rose according to the increase in the carbon source.

Hypoxic conditions in the chemostat, as well as in fed batch cultures, have been shown to significantly increase the specific productivity of recombinant protein in *K. phaffii* [27,30]. As this is not a fermentative yeast, under aerobic conditions, the main yeast product is still biomass. However, under limited oxygen conditions, lactate production was favored over biomass both in chemostat and in batches, with lactic acid yields up to five times higher than in the aerobic phase, as demonstrated in a previous study [21]. Parallel metabolomic analyses revealed that the reduced oxygen availability for the electron transport chain leads to higher NADH/ NAD+ ratios under hypoxia [22].

**Figure 4:**
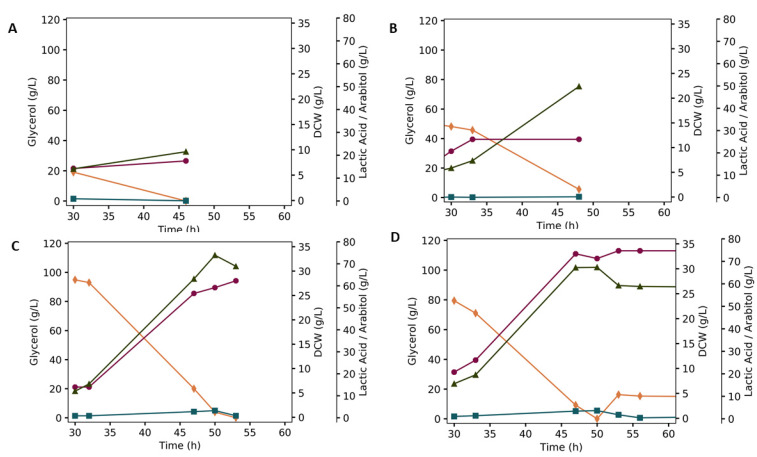
Fermentation profile for the GLp strain fed with different initial concentrations of glycerol in the restricted aerobic phase: 2% (**A**), 6% (**B**), or 8% (**C**). (**D**) GLpard strain, fed in the restricted aerobic strain with glycerol at 8%. Plotted are glycerol consumption (orange diamond), arabitol production (blue square), lactate production (green triangle) and dry cell weight (DCW) (purple circle). The experiments were carried out in biological duplicates, and the figure shows a representative fermentation profile.

In view of that, fed-batch fermentations were performed to evaluate lactic acid and arabitol production under oxygen limitation and different glycerol concentrations. In this set-up, during the first aerobic phase, dissolved oxygen was maintained at 50% and glycerol was added at 2% so biomass would be favored. In the second phase, the dissolved oxygen was limited to 5% and 2%, 6 % or 8% glycerol was added in a single pulse. Figure 4A–C shows only the restricted aerobic phase performed under different glycerol concentrations. 

When the bioreactor was fed with a 6% glycerol pulse (Figure 4B and Table 4), the best yield of lactic acid production using GLp as a biocatalyst was achieved, being approximately 85% from the theoretical yield. In addition to lactic acid, arabitol was detected as the largest co-product of this technological route with 7.2% yield. However, when we look at lactic acid productivity, there is a gradual increase as the glycerol increases with each fermentation. Therefore, it is feasible to increase the carbon source for greater lactic acid productivity.

It was previously hypothesized that in the GLp strain, as the PDC gene is interrupted in these strains, pyruvate is not able to restore the redox balance and this imbalance of cofactors would lead to DHAP for the pentose phosphate pathway, where arabitol would be formed by the reduction of ribulose or of xylulose through the consumption of NADH, which restores the redox balance of yeast [21].

An interesting issue is why, under low oxygen, the GLp strains reaches maximum yield at 6% glycerol, and a lower one at 10% (Table 4). While we still cannot account for this observation, we used this concentration to test whether the GLpard strain would show any improvement on GLp. As can be seen on Table 4, that was not the case: other than virtually eliminating arabitol, the GLpard strain showed an identical yield of 85% of lactic acid as its parental strain. Given that ArDH is hypothesized to be essential for restoring the redox balance caused by the fermentation of glycerol, deleting it would deprive the yeast of this route. This probably explains why there was no improvement on yield. A higher concentration of glycerol did not result in greater yield for GLpard (Figure 4D and Table 4). 

## 4. Conclusion

In this study, for the first time, a thorough investigation of metabolite formation was performed for a genetically modified strain of *K. phaffii* for lactic acid production using glycerol as substrate. Chemostat experiments were performed under aerobic and oxygen-limited conditions, corroborating that under respiratory metabolism, biomass is formed as a main product. Under aerobic conditions, lactic acid increased in yield concomitant to an increased dilution rate, which can be described as a Crabtree-like effect. Nevertheless, lactic acid was nearly five times higher under oxygen-limited conditions. Altogether, chemostast cultivations demonstrated an influence of both glycerol and oxygen concentration in the production of lactic acid. Surprisingly, no arabitol formation was observed during chemostat experiments, although batch and fed-batch fermentations also have shown a linear increase in arabitol formation with increased glycerol and reduced oxygen concentrations. This may be related to the lower lactic acid production under these conditions. Finally, deletion of arabitol dehydrogenase did significantly decrease arabitol formation. The residual arabitol we detected in the cultures indicates there are other dehydrogenases in *K. phaffii* that are still able to produce arabitol under oxygen-limited conditions. We also obtained significant improvements in yield by restricting glycerol to 6%, but that was in exchange for a lower productivity. This may indicate that the GLp strain has reached its optimal performance and the goal of increasing both productivity and yield will require further modifications. Hence, this study clarifies the underlying metabolic reasons for arabitol formation in *K. phaffii* and points to ways for improving production of lactic acid using *K. phaffii* as a biocatalyst.

## Figures and Tables

**Table 1:** Plasmids and strains used in this study.

Plasmids and Strains	Relevant Genotype	Ref.
pGEM^®^-T Easy	The pre-linearized Vector contains 3’-T protrusions at the insertion site to provide a compatible protrusion for PCR products.	Promega Corporation
pGEMHY ardh	*ArDH* 5′+Hygromyicin+ArDH3′	This study
pHIPH15 DsRed-SKL	*Ogataea polymorpha* vector with Hygromycin mark	[23]
GLp	GS115: *Δpdc* + pGAP-LDH Bos taurus	[21]
GLpard	GS115: *Δpdc + pGAP-LDH Bos taurus +Δardh*	This study

**Table 2:** Primers used in this study. Restriction enzyme sites are highlighted in bold.

Primers	Sequence (5′ 3′)	Restriction Site
Primer ArDH 5′ F	CGG**GCATGC**CCGATGTCGTTATCGTTAGACAACATAGTTCCA	SphI
Primer ArDH 5′ R	CGC**GGATCC**AGTATCCGGCGCAATGTACTAAG	BamHI
Primer ArDH 3′ F	TCC**CCCGGG**GAAATCCGGCCACAGAAACGG	SmaI
Primer ArDH 3′ R	CGG**ACTAGT**CCGTTACCAGCATTCGTAACCACCATCG	SpeI
Primer HIGRO 5′ F	CCG**GGATCC**CGCCCACACACCATAGCTTCAAAATGTT	BamHI
Primer HIGRO 3′ R	ATATCCC**CCCGGG**GGCGTTTTCGACACTGGATGGCG	SmaI

**Table 3:** Biomass, CO_2_, lactate, and arabitol yields (in Cmol/Cmol) and carbon recovery during steady-state of glycerol-limited chemostat cultures performed at different dilution rates (Dsp) and under two aeration conditions (% of oxygen in the inlet gas flow). Experiments were performed in duplicate for each steady-state and results are displayed as the average ± deviation of the mean.

	21% Oxygen			4% Oxygen
**Dilution rate (h^−1^)**	0.052	0.090	0.140	0.050
**Y_Biomass_**	0.736 ± 0.003	0.663 ± 0.023	0.689 ± 0.047	0.534 ± 0.023
**Y_CO_2__**	0.298 ± 0.003	0.269 ± 0.006	0.242 ± 0.027	0.190 ± 0.005
**Y_Lactate_**	0.007 ± 0.006	0.030 ± 0.007	0.049 ± 0.001	0.237 ± 0.016
**Y_Arabitol_**	0.000 ± 0.000	0.000 ± 0.000	0.000 ± 0.000	0.000 ± 0.000
**Glycerol** **Consumption rate (g.L^−1^.h^−1^)**	0.51 ± 0.0162	0.890 ± 0.0480	1.39 ± 0.0268	0.50± 0.0989
**C_Recovery_**	1.014 ± 0.006	0.984 ± 0.023	0.983 ± 0.020	0.964 ± 0.016

**Table 4:** Batch fermentation of GLp and GLpard strain performed under different oxygenation and glycerol (g) concentrations. Yields (Y) of biomass (x), lactate (lac), and arabitol are given in C-mol/C-mol ± the deviation of the mean. Experiments were performed in biological duplicates.

	Glycerol Concentration	Residual Glycerol (g/L)	Y_x/g_	Y_lac/g_	Y_ara/g_
**Oxygen 20%**	**GLp 2%**	0.982 ± 0.009	0.60 ± 0.130	0.370 ± 0.02	0.01 ± 0.008
	**GLp 6%**	1.81 ± 0.121	0.46 ± 0.018	0.200± 0.003	0.02 ± 0.010
	**GLp10%**	3.307 ± 0.231	0.37 ± 0.086	0.300 ± 0.055	0.12 ± 0.073
**Oxygen-limited**	**GLp2%**	0.608 ± 0.023	0.143 ± 0.037	0.559 ± 0.369	0.028 ± 0.000
	**GLp 6%**	1.16 ±0.008	0.087 ± 0.014	0.855 ± 0.077	0.012 ± 0.014
	**GLp 8%**	2.77 ± 0.044	0.239 ± 0.081	0.665 ± 0.025	0.072 ± 0.064
	**GLpard 6%**	1.975 ± 0.035	0.304 ± 0.067	0.8555 ± 0.66	0.004 ± 0.023
	**Glpard 8%**	2.134 ± 0.098	0.448 ± 0.013	0.763 ± 0.034	0.034 ± 0.015
